# Detecting parts-per-billion carbon monoxide with an ultra-enhanced near-infrared photoacoustic sensor

**DOI:** 10.1016/j.pacs.2025.100790

**Published:** 2025-12-18

**Authors:** Yaopeng Cheng, Ting Chen, Ruili Zhang, Sailing He

**Affiliations:** a Centre for Optical and Electromagnetic Research, College of Optical Science and Engineering, Zhejiang University, Hangzhou 310058, PR China; b Zhejiang Engineering Research Center for Intelligent Medical Imaging, Sensing and Non-invasive Rapid Testing, Taizhou Hospital, Zhejiang University, Taizhou 318000, PR China; c Zhejiang Key Laboratory of Digital Precision Measurement Technology Research, Zhejiang Institute of Quality Sciences, Hangzhou 310018, PR China; d National Engineering Research Center for Optical Instruments, Zhejiang University, Hangzhou 310058, PR China; e Department of Electromagnetic Engineering, School of Electrical Engineering, KTH Royal Institute of Technology, Stockholm SE-100 44, Sweden

**Keywords:** Near-infrared photoacoustic spectroscopy, Carbon monoxide, Hyperbolic nonlinear resonator, Near-concentric multipass cavity, Large-mode erbium-doped fiber amplifier

## Abstract

An ultra-enhanced near-infrared (NIR) photoacoustic gas sensor was developed by integrating three enhancing techniques: (a) boosting the excitation power up to 2 W via a custom-built large-mode erbium doped fiber amplifier (EDFA), (b) exploiting the acoustic resonance amplification of a hyperbolic nonlinear resonator (HNR), and (c) increasing the effective absorption path length by using a near-concentric multipass cavity (MPC) with 20 reflections. A weak CO absorption line at 1566.64 nm with the intensity of 2.074 × 10^−23^ cm/molecule was selected. The photoacoustic signal was enhanced 396 times. A minimum detection limit (MDL) of 190 ppb at 10 s was achieved and can be improved to be 11.4 ppb according to the Allan analysis, which was comparable to a mid-infrared (MIR) photoacoustic sensor. The ultra-enhanced NIR photoacoustic sensor is a cost-effective solution for the ppb-level trace gas detection, offering a price that is less than one-third that of MIR photoacoustic sensors.

## 1 Introduction

Carbon monoxide (CO) is a by-product of incomplete combustion of carbon-containing fuels, such as gasoline, natural gas, and coal that are widely used for petrochemical refining, transportation, power generation and other applications [1]. CO is colorless and odorless but highly toxic to humans. Exposure to high concentrations of CO can result in a range of adverse effects, including cardiac and pulmonary disorders, central nervous system involvement, fatigue, coma, and even death [2]. Furthermore, in atmospheric chemistry, CO is recognized as a major air pollutant due to its active role in tropospheric ozone formation and global warming [3], [4]. Therefore, the sensitive detection of CO is highly desired in monitoring emissions from carbon-containing fuels.

Photoacoustic spectroscopy (PAS) has been widely used in trace gas detection due to its high sensitivity and high selectivity [5], [6], [7]. Traditionally, PAS relied on microphones for the detection of acoustic waves. However, the quartz tuning fork (QTF) has recently emerged as a highly sensitive alternative. Consequently, the field has evolved from the use of conventional microphones to commercial QTFs [8], and more recently, to novel custom-designed QTFs [9] coupled with various quartz-enhancement techniques [10], [11], [12]. These advancements have substantially elevated the detection performance of the PAS-based sensors. The PAS-based CO sensors demonstrated minimum detectable limits (MDL) down to the ppb level using the quantum cascade lasers (QCLs) excite the strong fundamental rotation-vibration band (4.6 μm) in the mid-infrared (MIR) of CO [13], [14], [15], [16], [17], [18]. However, the price of QCLs is several thousand dollars [19]. Also, QCL has a large threshold current, and thus a high power consumption in the order of few watt [20]. Therefore, the operation of QCL requires an additional active air or water cooling to manage the substantial heat generated by the power consumption [21], which increases the overall size, weight, and power (SWaP) of the sensor.

Near-infrared (NIR) diode lasers are attractive alternative to the QCLs in the gas sensing application. Typical emission wavelengths of commercial NIR diode lasers are in the spectral range of 0.5–2 μm which covers the gas vibrational overtones spectra [22]. NIR diode lasers have long service life (> 10 years) and low cost [23]. They can be easily operated at room temperature without any active cooling, simplifying the entire system and reducing the SWaP of the sensor. This makes them more suited for practical, field-deployable applications. Their narrow linewidths (<1 MHz), wide tuning ranges and great modulation capability are ideal for high selective and sensitive trace gas detection. Typically, they come with pigtailed single-mode fibers, which facilitate the beam delivery and enable efficient beam coupling into different gas sensor configurations [24]. The output power of the NIR diode lasers is on the order of tens of milliwatt, but can be readily boosted up to the watt level by using telecommunication optical fiber amplifiers. However, the gas absorption line intensity in the NIR region is typically 2–4 orders of magnitude lower than those in the MIR region. Fortunately, various techniques for enhancing photoacoustic signals have been extensively developed and reported [23]. By strategically exploiting and integrating these techniques, the limitation of weak gas absorption in the NIR region can be effectively compensated.

In this paper, a NIR diode laser was employed for the trace CO detection. Three techniques to enhance the photoacoustic (PA) signal were adopted: (a) boosting excitation power via a custom-built large-mode erbium doped fiber amplifier (EDFA), (b) exploiting acoustic resonance amplification by a hyperbolic nonlinear resonator (HNR) with a high setup constant, and (c) increasing the effective absorption path length by using a near-concentric multi-pass cavity (MPC). The cost of developing an ultra-enhanced NIR PA sensor was less than one-third that of a MIR PA sensor. In trace CO detection experiments, an MDL of 190 ppb at 10 s was achieved and can be improved to be 11.4 ppb according to the Allan analysis.

## 2 Materials and methods

### 2.1 Selection of the CO absorption line

**Fig. 1:**
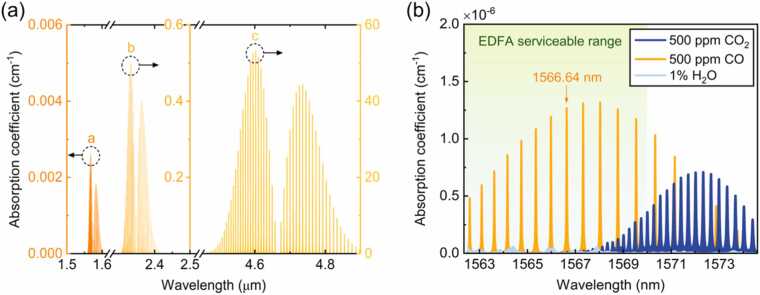
Selection of CO absorption line. (a) Three main infrared absorption bands of CO. (b) Absorption coefficients of 500 ppm CO, 500 ppm CO_2_ and 1 % H_2_O from 1562 to 1574 nm. All absorption coefficients are simulated at 1 atm and 296 K based on the HITRAN database.

As shown in Fig. 1(a), in the infrared region, the CO molecule has three main absorption bands: a. the second overtone band (1.56 μm), b. the first overtone band (2.33 μm), and c. the fundamental band (4.6 μm). In terms of the light source, NIR diode lasers are preferred over QCLs. Therefore, the two NIR absorption bands of CO (1.56 μm and 2.33 μm) are considered. The second overtone band (1.56 μm) of CO is two orders of magnitude weaker than the first overtone band (2.33 μm). However, it located at the wavelength window for optical fiber communication, so an optical fiber amplifier can be employed to boost the optical power. Hence, the second overtone band (1.56 μm) is a chosen to implement the NIR trace CO detection. The potential spectral interference from atmospheric water vapor (H_2_O) and carbon dioxide (CO_2_) was evaluated. As shown in Fig. 1(b), absorption coefficients of 500 ppm CO, 500 ppm CO_2_ and 1 % H_2_O were simulated based on the HITRAN 2020 database [25]. Accordingly, the CO absorption line at 1566.64 nm with the line intensity of 2.074 × 10^−23^ cm/molecule was selected in this work.

### 2.2 Techniques to enhance the photoacoustic signal

For resonant operation, the laser modulation frequency is tuned to one of the resonance frequencies of the PA cell. The PA signal $S_{\mathrm{PA}}$ measured by the microphone is given by [23]:

(1) $$\begin{array}{l} S_{\mathrm{PA}}=\frac{\left(\gamma -1\right)\mathrm{LQ}}{\omega V}P\alpha S_{\mathrm{mic}} \end{array}$$

where γ is the adiabatic coefficient of the gas, L is the absorption path length, Q is the quality factor of the PA cell, ω is the resonance frequency of the PA cell, V is the volume of the PA cell, P is the laser power, α is the gas absorption coefficient, and $S_{\mathrm{mic}}$ is the microphone sensitivity.

Eq. (1) indicates the three main techniques to enhance the PA signal: (a) boosting the laser power P by using an optical amplifier; (b) exploiting the acoustic resonance amplification by employing a resonant PA cell with a high quality factorQ; (c) increasing the effective absorption path length L by introducing an MPC that allows the laser beam to reflect multiple times within the PA cell.

### 2.3 Design of the HNR and the near-concentric MPC

**Fig. 2:**
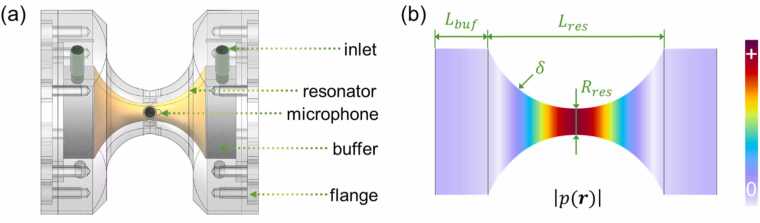
(a) A sketch of our hyperbolic nonlinear resonator. (b) Geometric features of the cavity and simulated distribution of the absolute pressure at resonance.

The nonlinear resonator, an axisymmetric closed tube with a varying cross section, exhibits better acoustic resonance effects than a traditional cylindrical resonator [26], [27], [28]. Therefore, a hyperbolic nonlinear resonator (HNR) was designed and used as a resonant PA cell to enhance the PA signal through acoustic resonance amplification. The sketch of the HNR is shown in Fig. 2(a), mainly including a hyperbolic resonator tube (colored in orange), two buffer chambers (colored in gray), gas inlet and outlet (colored in green), and four microphones. As shown in Fig. 2(b), finite element analysis (FEA) was performed based on the harmonic full linearized Navier–Stokes (FLNS) equations to simulate the pressure distribution in the HNR [29], [30]. A Gaussian distributed heat source with an amplitude of 0.35 W/m^3^ was introduced along the axis of symmetry of the HNR to simulate the gas absorption by a single-pass modulated laser beam. Through simulation, the *L*_*res*_, *R*_*res*_, *δ*, and *L*_*buf*_ of the HNR were optimized to 40 mm, 3 mm, 120 m^−1^, and 12 mm, respectively. The resultant HNR obtained a strong $\left|p\left(r_{\mathrm{mic}}\right)\right|$ of 1.83 × 10^−5^ Pa, leading to a high setup constant $c_{\mathrm{cell}}\;$ of 3420 Pa·cm/W.

**Fig. 3:**
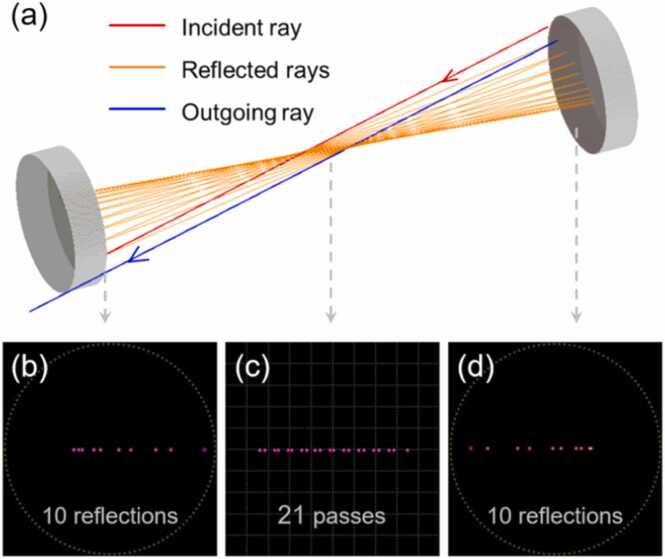
Simulation of the near-concentric multipass cavity. (a) Ray tracing. Irradiance maps of (b) the left concave mirror, (c) the center of the multipass cavity, and (d) the right concave mirror.

A near-concentric MPC was designed to further enhance the PA signal. The near-concentric MPC consisted of two silver-coated concave mirrors with a focal length of 25 mm and a diameter of 25.4 mm. The reflectance of the mirrors at a wavelength of 1566 nm was above 97.3 %. The two concave mirrors were coaxially positioned 100 mm apart to satisfy the near-concentric condition. The number of reflections was determined by the distance of incidence, the angle of incidence, and the tilt angle of the mirror. We simulated the optical path of the near-concentric MPC, with comprehensive details available in Appendix A. By adjusting these three parameters to 0.6 mm, 13.48°, and 0.19°, respectively, up to 20 reflections were achieved. The optical path and irradiance maps of the near-concentric MPC are shown in Fig. 3(a) and (b) respectively. The laser beam entered the MPC, reflected 10 times on each mirror, and then exited the MPC after 21 passes.

### 2.4 Design of the large-mode erbium-doped fiber amplifier

**Fig. 4:**
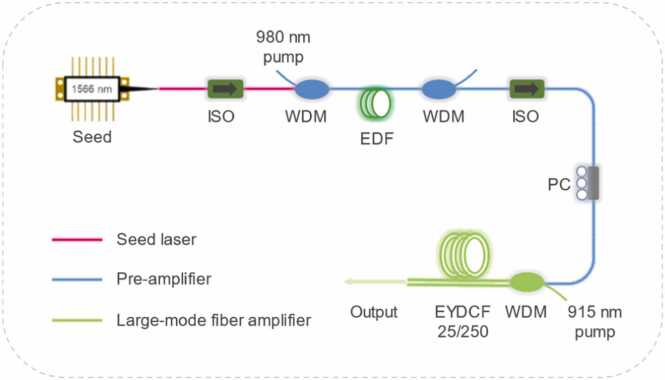
Configuration of the large-mode erbium-doped fiber amplifier. ISO: isolator; WDM: wavelength division multiplexer; EDF: erbium-doped fiber. EYDCF: large-mode-area Er/Yb co-doped double-cladding fiber.

A custom-built large-mode erbium-doped fiber amplifier (EDFA) is configured as Fig. 4. The amplifier system consisted of a pre-amplifier followed by a large-mode fiber amplifier. The optical isolators (ISOs) were employed to suppress back-reflection and ensure unidirectional operation, while wavelength division multiplexers (WDMs) employed for pump-signal combination. In the pre-amplifier, a ∼ 60 cm segment of commercial single-cladding erbium-doped fiber (LIEKKI, Er-110–4–125) was used as the gain medium. The EDF was bidirectionally pumped by a commercial 980 nm laser with a maximum output power of 600 mW. A polarization controller (PC) was placed before the large-mode fiber amplifier to optimize the signal polarization state. In the large-mode fiber amplifier stage, a ∼ 3.5 m segment of commercially available large-mode-area Er/Yb co-doped double-cladding fiber (EYDCF, IXF-2CF-EY-O-25–250-COM) was used as the gain medium. This fiber features a core diameter of 25 μm, which significantly reduces the accumulated nonlinear effects and thereby facilitates high-power laser generation [31]. A commercial WDM was utilized to simultaneously couple a 915 nm multimode laser diode pump into the cladding and the energized the seed laser into the core of the EYDCF. The 915 nm laser provided an adjustable pump power ranging from 0 to 25 W.

## 3 Experimental section

### 3.1 System configuration and sample gas preparation

**Fig. 5:**
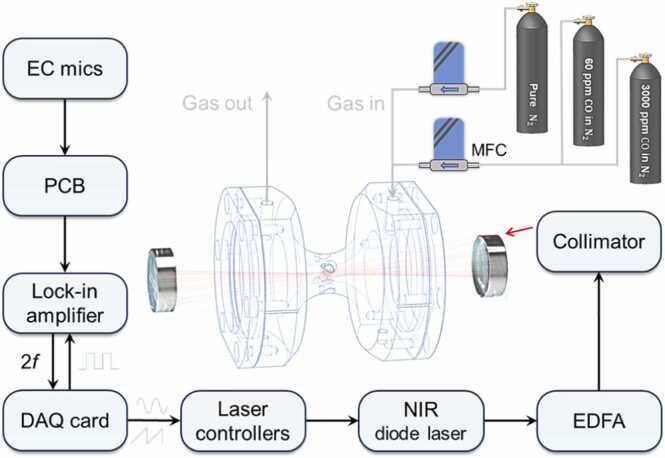
Simplified block diagram of the ultra-enhanced NIR PAS-based CO sensor. EC mic: electret condenser microphone; PCB: printed circuit board; 2 *f*: second harmonic signal; DAQ: data acquisition; EDFA: erbium doped fiber amplifier; MFC: mass flow meter.

A schematic configuration of the NIR CO sensor is depicted in Fig. 5. In our sensor, a 1566 nm distributed feedback laser (DFBL) with an optical power of 50 mW (OptoChip Optoelectronics, China, Model OC45650SMF) was chosen as the optical excitation source and was subsequently amplified by the custom-built large-mode EDFA to 2 W. The output laser beam was collimated by a fiber collimator (Thorlabs, USA, Model F230FC-1550) and coupled into the near-concentric MPC via a high-precision XYR translation stage. One mirror was mounted on a manual kinematic mount (Thorlabs, USA, Model KS1), while the second mirror was installed on a 2-axis piezo-driven mount (Thorlabs, USA, Model PIM1/M). The tilt angle of the mirror was finely controlled by a piezo inertia motor controller (Thorlabs, USA, Model KIM101), allowing for precise alignment to achieve 20 reflections. Wavelength modulation spectroscopy with second harmonic detection (WMS-2f) was adopted. A modulation signal composed of a sawtooth wave of 0.1 Hz and a sine wave of 3.232 kHz (half of the resonance frequency of the HNR) was sent to the laser controller to drive the DFBL. A temperature controller (Thorlabs, USA, Model TED200C) and a current driver (Thorlabs, USA, Model LDC205C) were employed to stabilize the laser temperature and convert the modulation signal into the laser driving current. To improve the acoustic detection performance, we used four microphones to detect the PA signal. The PA signal scales with the number n of the microphones, while the uncorrelated noise increases only by a factor of $\sqrt{n}$ [32]. Thus, the signal-to-noise ratio improves with $\sqrt{n}$. A homemade printed circuit board (PCB) amplified and summed the four microphone signals. The summed signal was connected to the lock-in amplifier (LIA, AMETEK, USA, Model 7270) to demodulate the second harmonic component (2 *f*). The equivalent noise bandwidth (ENBW) of the LIA was set to 0.33 Hz to reduce noise. A data acquisition card (DAQ, National Instruments, USA, Model USB-6229) along with a custom-written LabVIEW program were utilized to provide the modulation signal, generate the reference signal, collect the 2 *f* signal, and display the measured results. All the experiments were performed in the air-conditioned laboratory with the room temperature of 23℃. The gas preparation system consisted of two standard gas tanks of 60 ppm and 3000 ppm CO diluted in N_2_, a gas tank of pure N_2_, and two high-precision mass flow controllers (MFCs). Various CO/N_2_ gas mixtures of defined concentrations were prepared for detection experiments by means of MFCs. The standard gas sample of CO was flow-control diluted by the pure N_2_ via the two MFCs and the CO concentrations of gas mixtures were varied by changing the ratio of the flow rates of the two MFCs.

### 3.2 Characterization of the large-mode erbium-doped fiber amplifier

**Fig. 6:**
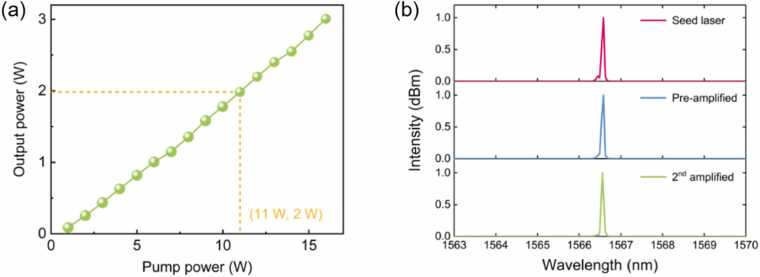
Performance characterization of the custom-built large-mode EDFA. (a) Output power at 1566 nm as a function of pump power. (b) Output spectra measured at different amplification stages.

The performance of the custom-built large-mode EDFA was systematically characterized. As illustrated in Fig. 6(a), the output power at 1566 nm as a function of the 915 nm pump power was investigated using an optical power and energy meter consoles (Thorlabs, USA, Model PM100D2). The amplifier exhibited a highly linear input-output relationship. The seed laser, with a power of 50 mW was first amplified to 162 mW by the pre-amplifier. Then the output power scaled efficiently from 162 mW to over 3 W by the large-mode fiber amplifier as the pump power increased from 1 W to 16 W. This linear trend indicated an excellent pump conversion efficiency and the absence of significant parasitic lasing or thermal roll-over within this operating range. An output of 2 W, generated at a pump power of 11 W, was employed for the subsequent gas sensing experiments.

The spectral evolution through the amplifier stages was recorded by an optical spectrum analyzer (Thorlabs, USA, Model OSA203C). Fig. 6(b) presents the optical spectra of the seed laser, after the pre-amplifier, and after the large-mode fiber amplifier. The center wavelength remained stable throughout the amplification stages, with no observable shift or broadening within the instrument resolution of 0.06 nm. Furthermore, the side-mode suppression ratio (SMSR) was maintained at a high level throughout the system, showing a slight increase from 47.0 dB for the seed laser to 49.5 dB after the pre-amplifier, and reaching 50.3 dB after the large-mode fiber amplifier. No severe spectral distortion or emergence of new spectral components was observed. The results demonstrated the amplifier's superb spectral stability and its linear operating regime, effectively suppressing excessive amplified spontaneous emission (ASE) and nonlinear spectral broadening.

### 3.3 Modulation optimization for the HNR

**Fig. 7:**
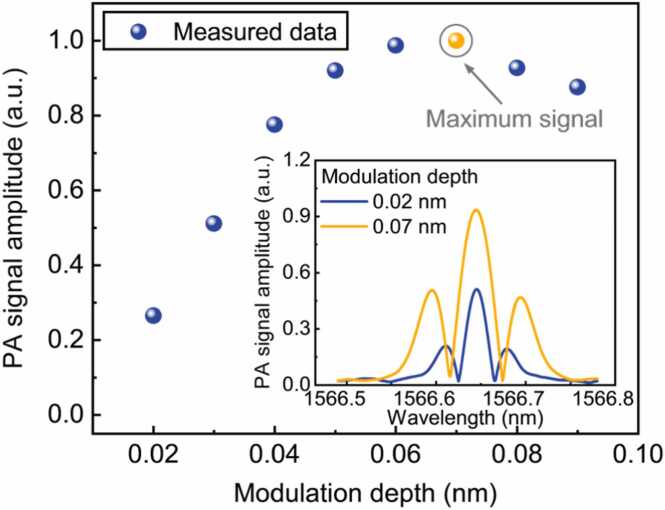
Photoacoustic 2 *f* signal peak amplitude as a function of the modulation depth for 3000 ppm CO/N_2_ at 1 atm and 296 K. Inset: representative PA 2 *f* curves at the modulation depths of 0.02 and 0.07 nm. The amplitude was normalized to one.

The laser modulation depth has a significant impact on the 2 *f* signal. Hence, the dependence of the 2 *f* signal amplitude on the modulation depth was investigated. Experiments were conducted with a certified CO/N_2_ concentration of 3000 ppm. The modulation frequency was set to half of the resonance frequency of the HNR (3.232 kHz). The peak values of the 2 *f* signals were recorded as modulation depths varied from 0.02 to 0.09 nm with an increment of 0.01 nm. As shown in Fig. 7, with increasing modulation depths, the amplitude of the 2 *f* signal first increased, peaked at the modulation depth of 0.07 nm, and then decreased. Two representative PA 2 *f* curves at the modulation depths of 0.02 and 0.07 nm are illustrated in the inset graph of Fig. 7. Accordingly, the modulation depth of 0.07 nm was selected in this work to achieve the strongest 2 *f* signal amplitude.

### 3.4 Enhancement of the photoacoustic signal

**Fig. 8:**
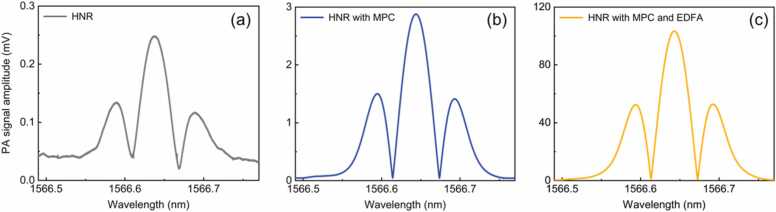
Photoacoustic 2 *f* curves for 3000 ppm CO/N_2_ at 1 atm and 296 K measured by three different configurations: (a) bare hyperbolic nonlinear resonator (HNR), (b) HNR with the near-concentric multi-pass cavity (MPC), and (c) HNR with the near-concentric MPC and custom-built large-mode EDFA.

We tested the effectiveness of the three adopted signal-enhancing techniques. Experiments were conducted under the same experimental conditions for fair comparisons. The 2 *f* signal curve was recorded in the standard 3000 ppm CO/N_2_ with three different system configurations: (a) the single-pass HNR; (b) the multi-pass HNR; (c) the multi-pass HNR with the power boosted by the EDFA. The results are shown in Fig. 8. The 2 *f* signal peak amplitude of the multi-pass HNR was 2.88 mV, which was approximately 11 times stronger than that of the single-pass HNR (0.248 mV). As for the enhancement by EDFA, the 2 *f* signal peak amplitude of the multi-pass HNR with the power boosted was up to 103 mV, resulting in a 36-fold signal enhancement than that of the multi-pass HNR (2.88 mV). By adopting these techniques, the PA signal was enhanced by a factor of 396 altogether; the weak CO gas absorption line strength in the NIR region was compensated. An increase in baseline level, from 0.042 mV to 0.12 mV, was observed. This rise is due to the use of the near-concentric MPC and the EDFA, which can contribute to a higher background PA signal. The enhancement by the near-concentric MPC can be further improved by increasing the number of reflections, the reflectance of the concave mirrors, and the transmission of the optical windows of the HNR, while the enhancement by the EDFA can be improved by simply increasing its output power.

### 3.5 Photoacoustic detection of CO diluted in N_2_

**Fig. 9:**
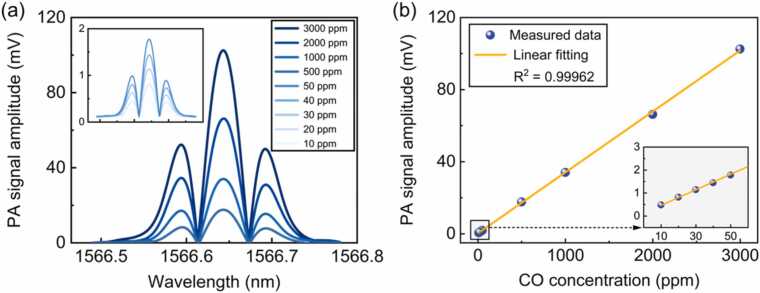
The calibration of the ultra-enhanced NIR PA sensor for the detection of CO at 1 atm and 296 K. (a) Measured 2 *f* signal curves at different CO concentrations. (b) Photoacoustic 2 *f* signal peak amplitude as function of CO concentration.

The concentration response of the developed PA sensor was examined through the calibration. A series of CO/N_2_ gas mixtures of known concentrations from 10 to 3000 ppm were successively introduced into the PA cell for the sensor calibration. Fig. 9(a) depicts the measured PA 2 *f* signal curves at different concentrations of CO. The corresponding peak values of the 2 *f* signals are plotted in Fig. 9(b) as a function of CO concentration. According to the linear fit, the sensor showed an excellent linear response to CO concentrations in the range of 10–3000 ppm with an R-square value of 0.99962 and a calibration coefficient of 0.0338 mV/ppm.

**Fig. 10:**
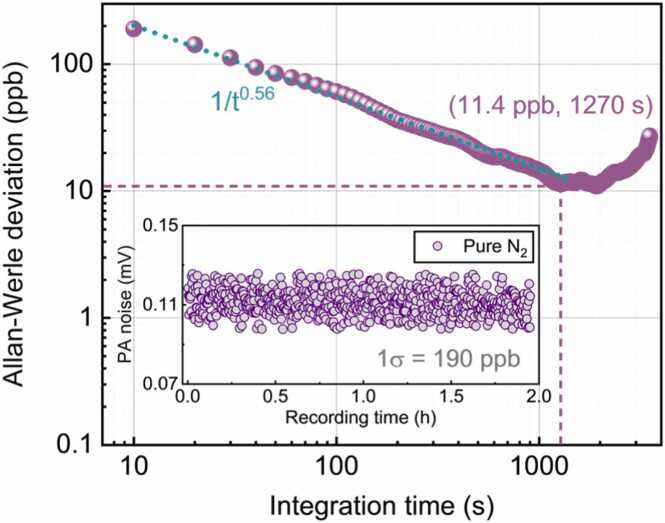
The Allan–Werle deviation analysis. The dashed line represents the 1/*t*^0.56^ slope. Inset: the raw data of the PA 2 *f* noise measured in pure nitrogen for 2 h.

As a common tool to demonstrate the stability performance of the sensor, Allan–Werle deviation analysis was performed to further investigate the long-term stability and optimal achievable MDL of the developed NIR PA sensor. The pure N_2_ was continuously flushed into the PA cell at a flow rate of 300 mL/min, and the 2 *f* noise was collected every 10 s without averaging for approximately two continuous hours. As shown in the inset of Fig. 10, with the integration time of 10 s, the MDL was tested to be 190 ppb. The Allan–Werle deviation analysis was then performed on the 2 *f* noise in N_2_ and the results are shown in Fig. 10. The deviations in millivolt units were converted into concentration units (ppb) using the calibration coefficient of the sensor. As the integration time was extended and the white noise was suppressed, the Allan–Werle deviations decreased as the function of 1/*t*^0.56^. The MDL can be improved to 11.4 ppb with an integration time of 1270 s. According to the Allan–Werle analysis, the developed PA sensor exhibited excellent MDL performance and long-term stability.

Table 1 shows the comparison between the developed ultra-enhanced NIR PA CO sensor and previously reported PAS-based CO sensors. In the NIR region, our ultra-enhanced NIR PA sensor achieved the best MDL of 11.4 ppb, which was comparable to that obtained by MIR PA sensors. However, the cost of the ultra-enhanced NIR PA sensor was merely one-third that of the MIR PA sensors. In the NIR region, reference [33], [34] also utilized the EDFA to boost the laser power. However, they all were based on a traditional H-type PA cell and operated without a multi-pass cavity and they obtained less competitive MDLs than our ultra-enhanced NIR PA sensor.

**Table 1:** Comparison of the developed ultra-enhanced NIR PA CO sensor and previously reported PAS-based CO sensors. MDL: minimum detectable limit. PAS: photoacoustic spectroscopy; HPAS: heterodyne photoacoustic spectroscopy; QEPAS: quartz enhanced photoacoustic spectroscopy; DFBL: distributed feedback laser; QCL: quantum caseate laser.

Band	Methods	Target lines	Laser	Power	MDL
MIR	HPAS	4.61 μm	QCL	21 mW	26 ppb [13]
	PAS	4.61 μm	QCL	21 mW	8 ppb [14]
	QEPAS	4*.*57 μm	QCL	32 mW	12 ppb [16]
	QEPAS	4.59 μm	QCL	40 mW	22 ppb [18]
	QEPAS	4.65 μm	QCL	71 mW	2 ppb [17]
	PAS	4.60 μm	QCL	175 mW	0.8 ppb [15]
NIR	QEPAS	2.33 μm	DFBL	3.3 mW	1.3 ppm^a^[35]
	CEPAS	2.33 μm	DFBL	Not given	5.1 ppm [36]
	PAS	1.568 μm	DFBL	1 W*	0.47 ppm^b^[34]
	PAS	1.566 μm	DFBL	10 W*	110 ppb [33]
	PAS	1.566 μm	DFBL	2 W*	11.4 ppb [**This work**]

* : amplified by EDFA. a: H_2_O as the promotor; b: sulfur hexafluoride (SF_6_) as the promotor.

## 4 Conclusion

In conclusion, a NIR diode laser was employed for the trace CO detection. Three techniques to enhance the PA signal were adopted: (a) boosting excitation power up to 2 W via the custom-built large-mode EDFA, (b) exploiting acoustic resonance amplification by the HNR and (c) increasing the effective absorption path length by using the near-concentric MPC with 20 reflections. By adopting these techniques, the PA signal was enhanced by a factor of 396 altogether. In trace CO detection experiments, the ultra-enhanced NIR PA sensor compensated the weak CO absorption line strength in the NIR spectral region. An MDL of 190 ppb at 10 s was achieved and can be improved to be 11.4 ppb according to the Allan analysis, which was comparable to that obtained using an MIR PA sensor. The ultra-enhanced NIR PA sensor was a cost-effective solution for the ppb-level detection of trace gases, offering a price point that is less than one-third that of MIR PA sensors. The present PA gas sensing system can also be utilized for other applications such as fast and accurate measurement of exhaled breath gases for medical applications In medicine, the concentration of CO in exhaled human breath is analyzed as an indicators of health and for the diagnosis of certain ailments, such as asthma, anemia and inflammation [37], [38].

## CRediT authorship contribution statement

**Ting Chen:** Visualization, Software, Investigation, Data curation. **Yaopeng Cheng:** Writing – original draft, Visualization, Validation, Software, Methodology, Data curation, Conceptualization. **Sailing He:** Writing – review & editing, Supervision, Resources, Project administration, Funding acquisition, Formal analysis. **Ruili Zhang:** Supervision, Resources, Investigation, Formal analysis.

## Declaration of Competing Interest

We declare that we have no financial and personal relationships with other people or organizations that can inappropriately influence our work. We also declare no conflicts of interests with each other on this study.

## Figures and Tables

**Fig. A1:**
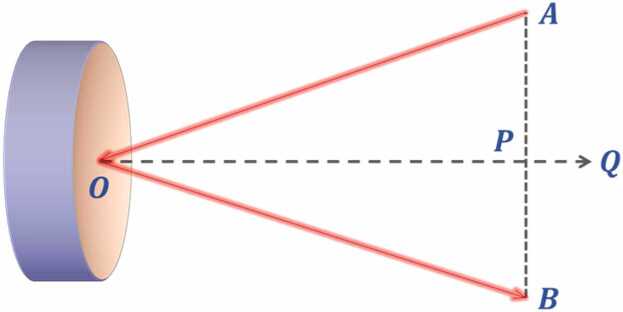
Schematic diagram of ray tracing model: to calculate the direction vector of the reflected ray

## Data Availability

Data will be made available on request.

## Appendix A Simulation of near-concentric multipass cavity

The number of reflections in the near-concentric multipass cavity (MPC) is determined by three key parameters: the incident distance (distance of the beam from the optical axis at the entrance mirror), the incident angle, and the tilt angle of the mirror. To design the near-concentric MPC, we developed a ray-tracing program in MATLAB. First, the theoretical upper limit for the number of reflections (*N*) within the cell was estimated from:

(1) $$\begin{array}{l} N=\frac{2\arcsin\left(D/4f\right)}{\arcsin\left(r/4f\right)} \end{array}$$

where D and f denote the diameter and focal length of the spherical mirror, respectively, and r is the radius of laser beam.

Next, the simulation explicitly models the reflecting surface of the spherical mirror via a three-dimensional surface function, incorporating key geometric parameters: the radius of curvature (focal length), physical aperture, and tilt angle relative to the optical axis. The incident laser beam is defined by a ray equation characterized by its coordinate of origin (incident distance) and direction vector (incident angle). And, the reflection point on the mirror is obtained by solving the intersection between the incident ray and the mirror surface.

Once the reflection point is known, the direction of the reflected ray is calculated using vector analysis. As illustrated in Fig A1, let $\vec{\mathrm{AO}}$ be the unit direction vector of the incident ray and $\vec{\mathrm{OQ}}$ be the unit surface-normal vector at the point of incidence, then the unit direction vector of the reflected ray $\vec{\mathrm{OB}}$ is given by:

(2) $$\begin{array}{l} \vec{\mathrm{OB}}=\vec{\mathrm{AO}}-2\left(\vec{\mathrm{AO}}∙\vec{\mathrm{OQ}}\right)\vec{\mathrm{OQ}} \end{array}$$

This vector calculation is iterated to trace the laser beam as it undergoes multiple reflections between the two mirrors. Through simulation, the near-concentric MPC was optimized by fine-tuning the incident distance, incident angle, and mirror tilt angle. By adjusting these three parameters to 0.6 mm, 13.48°, and 0.19°, respectively, we obtained 20 reflections in the near-concentric MPC.
